# Spatial analysis of provincial and district trends in stunting among children under five years in Nepal from 2001 to 2016

**DOI:** 10.1186/s40795-022-00629-1

**Published:** 2022-11-14

**Authors:** Sajama Nepali, Padam Simkhada, Balaram Thapa

**Affiliations:** 1 Local Initiative for Biodiversity Research and Development (LI-BIRD), PO Box 324, 33700 Pokhara, Nepal; 2 School of Human and Health Sciences, University of Huddersfield, Huddersfield, UK

**Keywords:** Children, District, Inequalities, Province, Spatial, Stunting

## Abstract

**Background:**

The average prevalence of stunting reported by the Nepal Demographic Health Survey from 2001 to 2016 only reports the prevalence of stunting at the national level and provincial and district level information is missing. Also, no previous study has reported a provincial trend in stunting from 2001 to 2016 in Nepal. This study for the first time presents the spatial trend of stunting among children under five years for 7 provinces and 77 districts of Nepal over 15 years using Demographic Health Survey Global Positioning System coordinates, Demographic Health Survey indicators, and geospatial covariates.

**Methods:**

This is a secondary analysis of data from Nepal Demographic Health Survey from 2001 to 2016. The study population was children under five years. The outcome variable was stunting, which was analyzed as per districts and provinces. Sample weight was applied to calculate the percentage of stunting and 95% confidence interval for all survey years. The geographic dataset was used to provide information about the latitude and longitude of the survey cluster. Poisson-based model was used during the purely spatial analysis in SatScan for identification of clusters with stunting caseload.

**Results:**

The reduction in the prevalence of stunting among children under five years has not been equal when disaggregated for district and provincial level data. In 2001, 57 districts had a prevalence of stunting among children above or equal to 50%, which has reduced over time except for districts in Karnali province. In 2016, 16 districts had a prevalence of stunting above or equal to 50%. Jumla (91.7%) and Kalikot (77.8%) still had the highest prevalence of stunting as of 2001. Among 7 provinces, the prevalence of stunting is found highest in Karnali for all subsequent survey years. Sudurpaschim and Madhesh provinces also had a high proportion of stunted children. The highest reduction in the prevalence of stunting was noted for Province Bagmati (by 30%) and Gandaki (by 28%).

**Conclusion:**

The inequalities in childhood stunting persisted at the district and provincial levels although a good decline was noted at the national level. This calls for rigorous attention to be provided to districts and provinces with a high prevalence of stunting, and being prioritized for a targeted intervention.

## Background

Stunting is a major public health problem in Nepal. The rate of stunting in Nepal is one of the highest in the world [1]. It occurs during the first thousand days of life, starting from conception to the second birthday. The immediate causes of stunting are insufficient food intake and infections such as diarrhea. Inadequate care, lack of health services, and socio-economic determinants are other causes of stunting [2]. The effects of stunting during the first 1,000 days are largely irreversible [3].

Globally, 149 million children under five years are stunted in 2020 [1]. The neighboring countries of Nepal also have a similar level of prevalence of stunting among children under five years. The prevalence of stunting among children in India and Pakistan was 38% [2] and 37.6% [3] as per respective DHS reports in 2015/16 and 2017/18. Nepal made an impressive progress in reducing the average prevalence of stunting with 21.4% reduction in 15 years, from 57.2% to 2001 to 35.8% in 2016 [1]. In regard to this, a lot of previous studies have identified the socio-demographic and economic determinants of stunting using Demographic Health Surveys (DHS) for understanding the significant determinants. So far, no studies have done spatial analysis on the trend of stunting at district and provincial levels for Nepal. A recent study by Nepali et al. has analyzed the trend and inequalities in stunting across socio-demographic and economic sub-groups using Nepal DHS from 2001 to 2016, but it lacks spatial information on districts and provinces [1].

The average prevalence reported by the study largely limits reporting the inequalities preserved in districts and provinces in Nepal. Understanding the differences at provincial and district levels is important. It is hypothesized that the prevalence of stunting among children under five years would be unequal when the national average would be separated into 77 districts and 7 provinces. The spatial presentation of trends of stunting in Nepal from 2001 to 2016 would be effective to highlight the provinces and districts with high and low prevalence crucial for prioritization of health resources and reducing child health inequalities. Considering this gap, the study for the first time presents the spatial trend of stunting among children under five years for 7 provinces and 77 districts of Nepal over 15 years using DHS Global Positioning System (GPS) coordinates, DHS indicators, and geospatial variable.

## Methods

### Data source

The data from Nepal Demographic Health Survey (NDHS) from 2001 to 2016 were used for this study. The NDHS for 2022 is ongoing and the report has not been published. Therefore, no information is available beyond 2016 in this study. NDHS is a cross-sectional study conducted every five years in all districts of Nepal. The NDHS provides an inclusive outline of population, maternal, and child health issues in Nepal to support policymakers and program managers in the Ministry of Health and other organizations in designing and assessing programs and approaches for refining the health of the overall nation’s population [4].

### Sampling

A two-stage sampling method was applied in NDHS from 2001 to 2011 and three-stage sampling method was used in NDHS 2016. The detail of the sampling is given in respective NDHS reports [4–7].

### Study area

Nepal has an area of 147,516 km^2^, lies in the foothills of Himalayas from 26˚22’ to 30˚27’ north latitude and 80˚4’ to 88˚12’ east longitude, with elevations ranging from 90 to 8848 m [8]. A landlocked nation with China’s Tibet to the north and surrounded by India on other three sides, Nepal is a sovereign, secular, federal democratic republican state, which was classified into 7 provinces on 20 September 2015 in accordance with schedule 4 of the Constitution of Nepal [9]. The classification split Rukum and Nawalparasi districts adding two more districts from a total of 75 to 77 districts. However, the study result is based on 75 districts since the split was not done during survey. Rukum was split into Rukum east and Rukum west, which were included in Karnali Province and Nawalparasi was split into Nawalparasi and Nawalpur, which were included in Lumbini Province in NDHS 2016. No provincial level information was available for NDHS 2001 to 2011. Therefore, the same classification of provinces was used for all survey years from 2001 to 2016 in this study for uniform unit of analysis.

### Study population

The study population was children under five years old and thus, a household member (PR) dataset was used, to match the result of this study with that of NDHS reports. The total sample size analyzed in this study was 16,606 (2001: 6442, 2006: 5258, 2011: 2485, 2016: 2421) for the four surveys, with a response rate of 96.1%, 96%, 95.3% and 95.9% for the year 2001, 2006, 2011 and 2016 respectively.

### Study variable

The outcome variable is stunting. Stunting is defined by World Health Organization (WHO) as the percentage of children aged 0 to 59 months, whose height for age is below − 2.00 to − 2.99 standard deviation (SD) for moderate and − 3.00 SD for severe stunting from the median of the 2006 WHO Child Growth Standards [2]. Stunting was classified as yes or no (a dummy variable) during transformation to geospatial variables during spatial analysis.

The independent variables were provinces and districts. As mentioned above, there are 7 provinces; (i) Province 1, (ii) Madhesh (iii) Bagmati (iv) Gandaki (v) Lumbini (vi) Karnali. and (vii) Sudur Paschim, and 77 districts in Nepal.

### Tools

DHS core questionnaires were contextualized for the Nepalese population. The data collection was done by a face-to-face method. The details of the tools are mentioned in respective NDHS reports.

### Statistical analysis

Data analysis was done using Microsoft Excel, R [10] and SPSS version 20 (IBM USA). The spatial analysis was done in Quantum Geographical Information System (QGIS) 3.22.4 [11] and SatScan [12]. The DHS sampling design includes both under and over-sampling, hence, all analyses were conducted with sample-weighted data [13]. Sample weight was applied to calculate the percentage of stunting and 95% confidence interval (CI) for all survey years.

The geographic dataset was used to provide information about the latitude and longitude of the survey cluster. However, the geographic dataset doesn’t include information about the household interviewed for maintaining the confidentiality of the respondents. Therefore, the analysis was done at the cluster level for Satscan analysis. The urban cluster coordinates were randomly displaced by 2 km whereas the rural cluster coordinates by 5 km in order to protect the confidentiality of participants [14]. The geographic dataset was used for the identification of clusters with stunting caseload during analysis in SatScan. The under-five population count from the respective survey years was used during the analysis in SatScan. There were 251 clusters in the 2001 survey year and 383 clusters in the 2016 survey year. Poisson-based model was used during the purely spatial analysis in SatScan for the identification of clusters with the stunting caseload. The maximum cluster size was set at 50% of the population at risk. Cluster restrictions were applied to have at least two cases followed by relative risk greater than or equal to 10.

## Ethics

Nepal Health Research Council approved the studies in Nepal. At the international level, since ICF Macro provided technical assistance, ICF Institutional Review Board approved all the NDHS. Similarly, the data collection tools and procedures for NDHSs were reviewed by the Independent Review Boards of New Era and ICF Macro International. The personal identifiers were removed from the data for anonymous results. Therefore, it was accessed through the DHS program website upon request and submission of the proposal noting the use of the dataset.

## Results

### Spatial analysis of trend of stunting among children under five years as per districts from 2001 to 2016

**Fig. 1:**
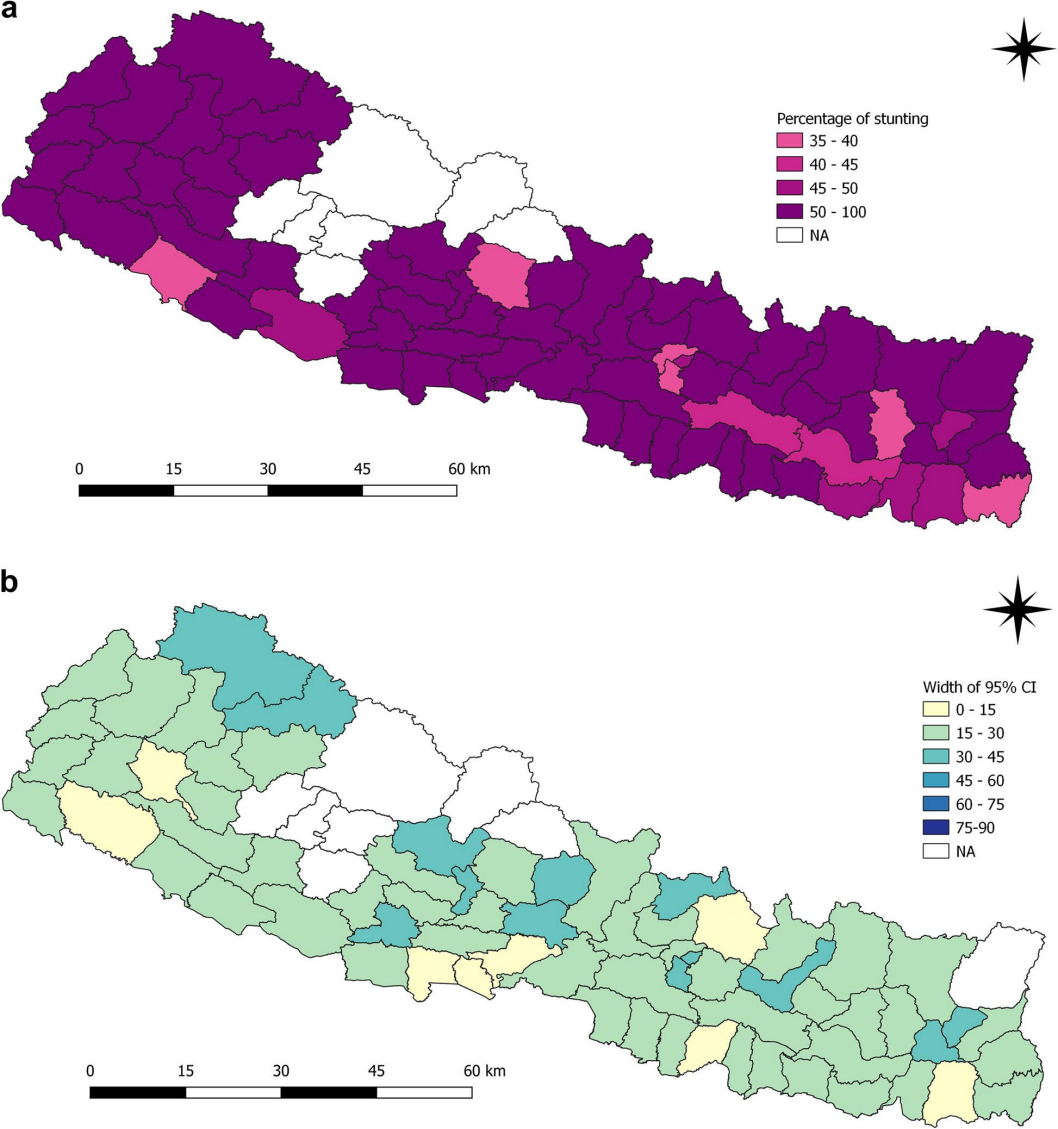
**a** and **b** Prevalence of stunting and width of 95% CI among children under five years as per districts in 2001

The average prevalence of stunting was 57.2% (95%CI 55.9–58.0) in 2001, 49.3% (95% CI 47.7–51.0) in 2006, 41.0% (95%CI 38.2–43.0) in 2011 and 35.8% (95%CI 33.7–38.0) in 2016 at national level. Marking a tremendous improvement, the prevalence was reduced by 21.4% in 15 years. The reduction has not been equal when disaggregated for districts and provincial level data. In 2001, 57 districts had prevalence of stunting above or equal to 50%, which has reduced over time except for districts in Karnali province (Fig. 1a and b). Dailekh (75.2%), Humla (90.0%), Jumla (76.9%), Arghakhanchi (71.1%), Bajura (79.8%), Dhading (74.0%), Kapilbastu (70.2%), Surkhet (70.4%) and Kalikot (75.0%) had the highest prevalence in 2001. Bhaktapur, Dang, Morang, Saptari, Sindhuli, Sunsari and Udaypur had prevalence between 40 and 50%. Similarly, Bardiya, Bhojpur, Jhapa, Kaski, Kathmandu and Lalitpur had prevalence between 30 and 40%.

**Fig. 2:**
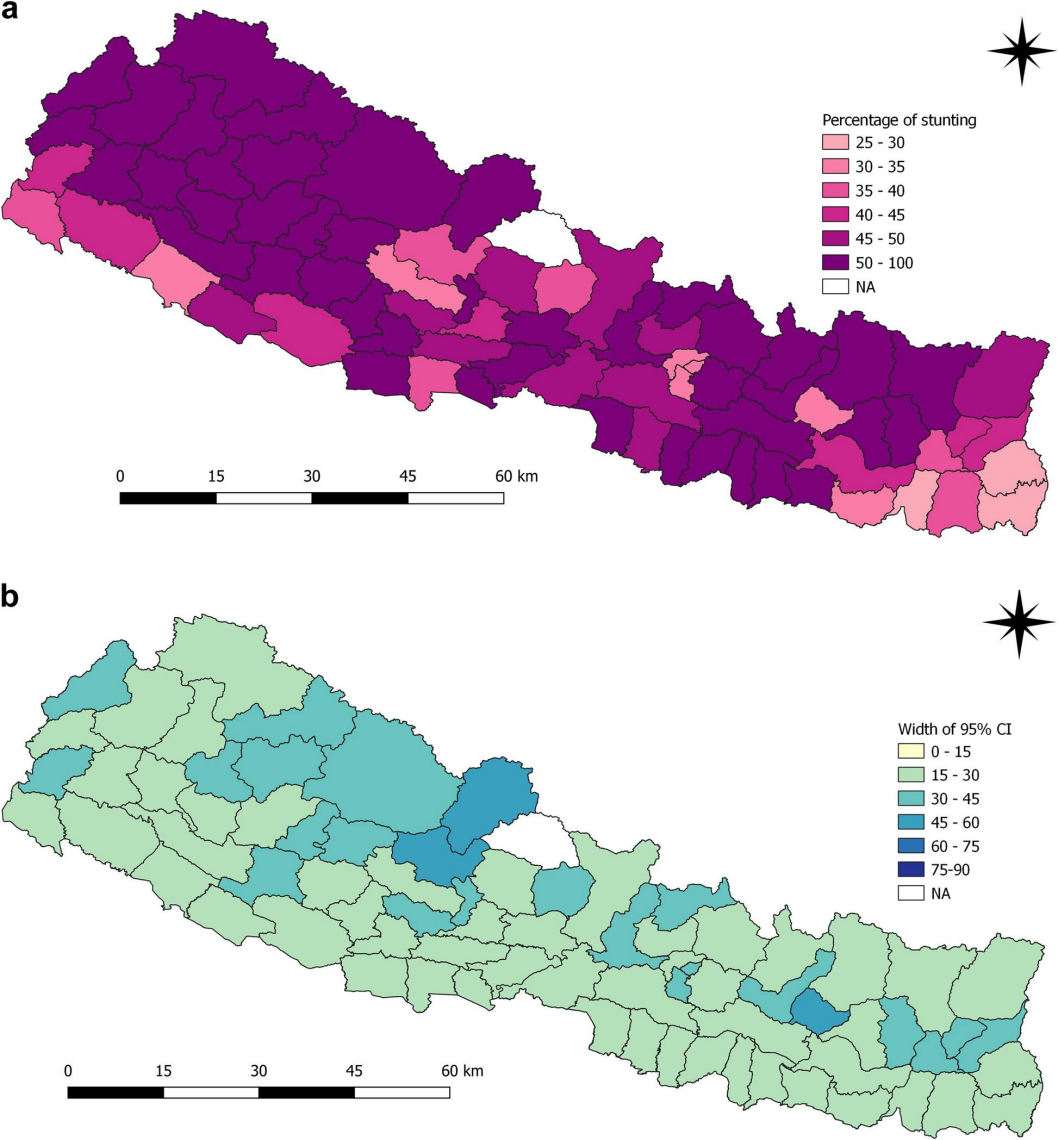
**a** and **b** Prevalence of stunting and width of 95% CI among children under five years as per districts in 2006

**Fig. 3:**
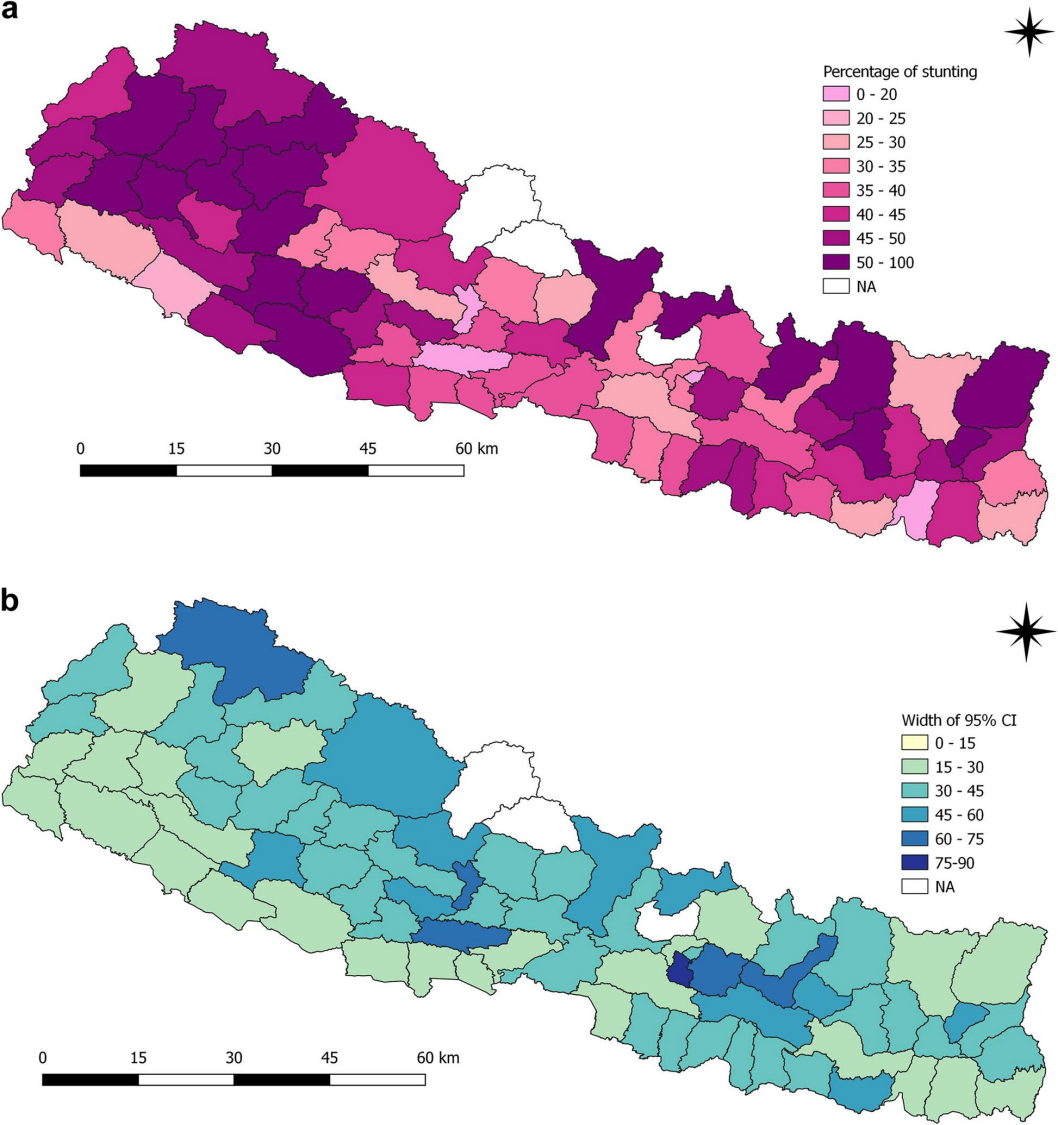
**a** and **b** Prevalence of stunting and width of 95% CI among children under five years as per districts in 2011

Districts such as Humla (88.2%), Rolpa (80.9%), Dailekh (71.7%), Arghakhanchi (71.1%), Mustang (75.0%), Bajura (73.5%), Salyan (69.2%) Accham (69.2%) and Bajhang (67.1%) had highest prevalence of stunting among children under five years in 2006 (Fig. 2a and b). In 2011, the districts with highest prevalence of stunting among children under five years were Achham (67.4%), Bajura (75.0%), Dolakha (66.7%), Jajarkot (75.0%), Jumla (81.3%) and Mugu (69.2%) (Fig. 3a and b).

**Fig. 4:**
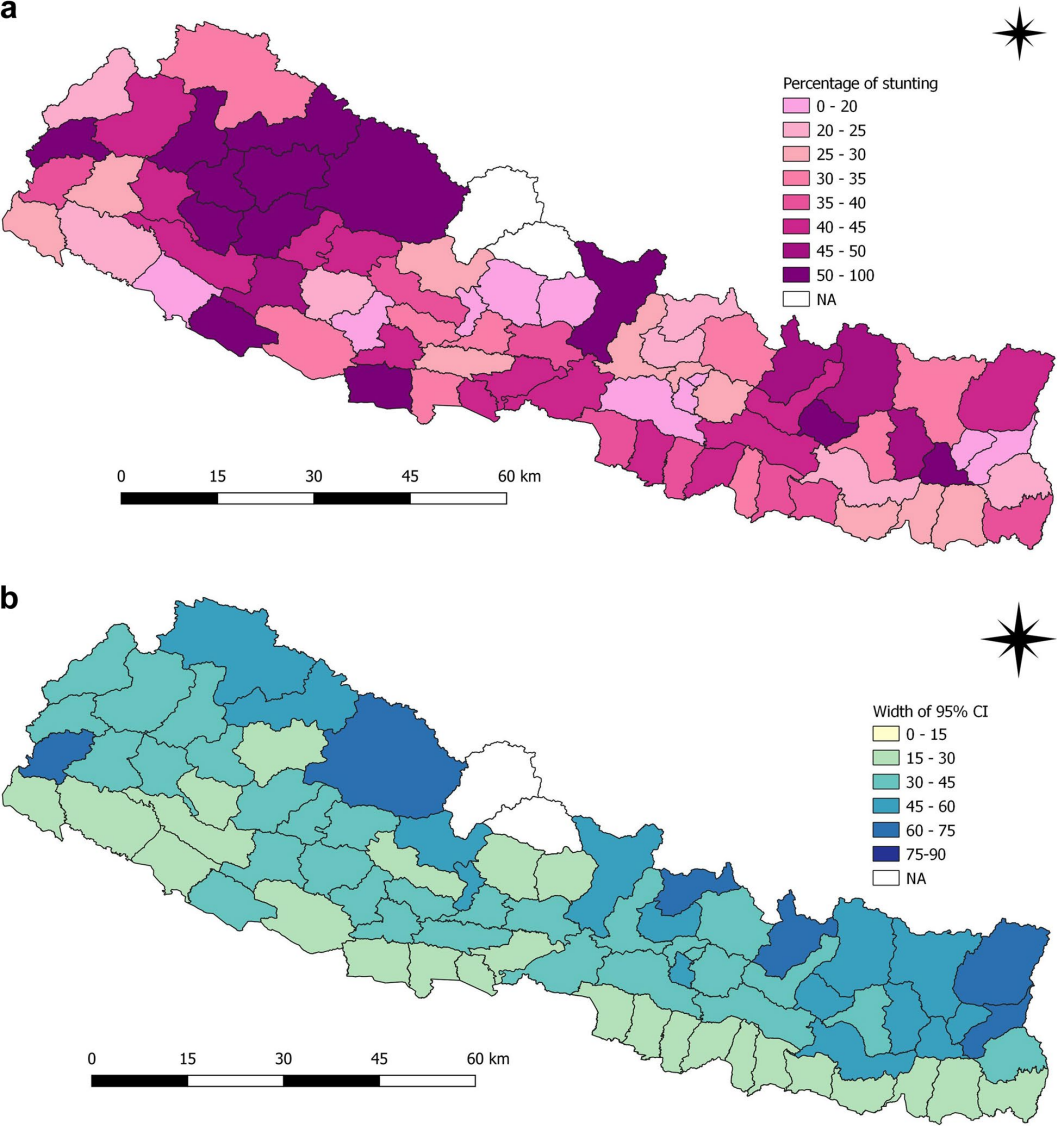
**a** and **b** Prevalence of stunting and width of 95% CI among children under five years as per districts in 2016

In 2016, 16 districts had prevalence above or equal to 50% (Fig. 4a and b). Jumla (91.7%) and Kalikot (77.8%) still had the highest prevalence of stunting as of 2001. Infact, their prevalence of stunting among children rose by 14.8% and 2.8% respectively. The prevalence of stunting among children in Bajura, Dailekh, Dolpa, Gorkha, Kapilbastu districts were 66.7%, 62.5%, 66.7%, 62.5% and 64.3% respectively. Besides, some other districts such as Banke, Bhojpur, Dolpa and Gorkha also reported an increase in the prevalence of stunting by 3.5%, 14.5%, 4.8% and 6.6% respectively over 15 years. Eighteen districts had prevalence between 40 and 50%. There were 19 districts with prevalence in between 30 and 40%. Rest of the districts were below 30%.

**Fig. 5:**
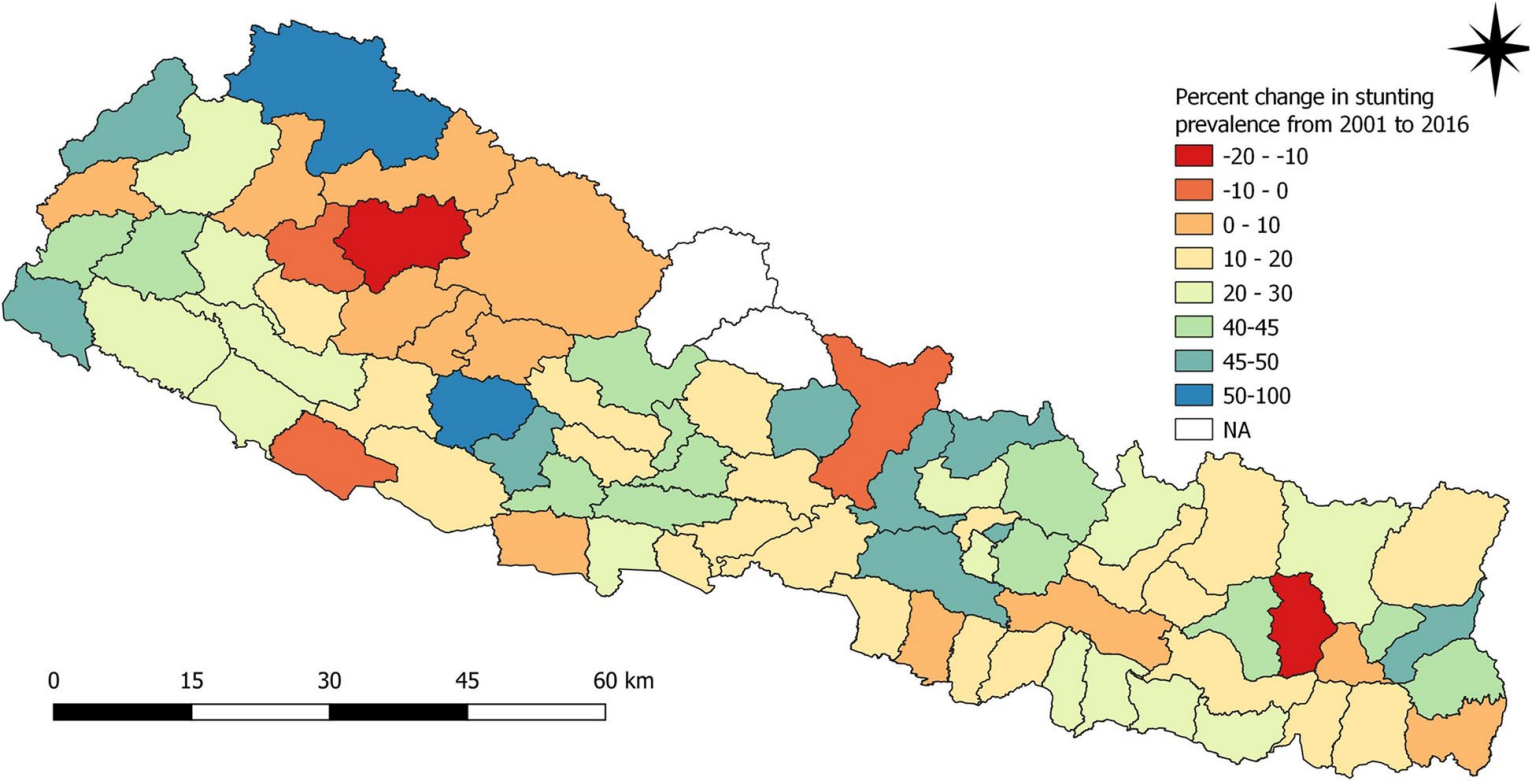
Percent change in prevalence of stunting among children under five years as per districts from 2001 to 2016

In contrary to this, a huge decline of above 40% in prevalence of stunting was noted for districts such as Bhaktapur, Dhading, Makwanpur, Rasuwa, Lamjung, Parbat, Pyuthan, Rolpa, Humla and Darchula over 15 years (Fig. 5). Figure 5 provides information about the percent change in prevalence of stunting among children under five years from 2001 to 2016. Data were not available for some districts (Dolpa, Jajarkot, Manang, Mustang, Rolpa, Rukum East and Rukum West districts) for 2001. In such a situation, the difference in prevalence of stunting was calculated for 2006 to 2016. Similarly, for 2006, data was not available for Manang. Data were not available for Mustang, Manang and Nuwakot districts for 2011 and for Manang and Mustang districts for survey year 2016.

### Spatial analysis of trend of stunting among children under five years as per provinces from 2001 to 2016

**Fig. 6:**
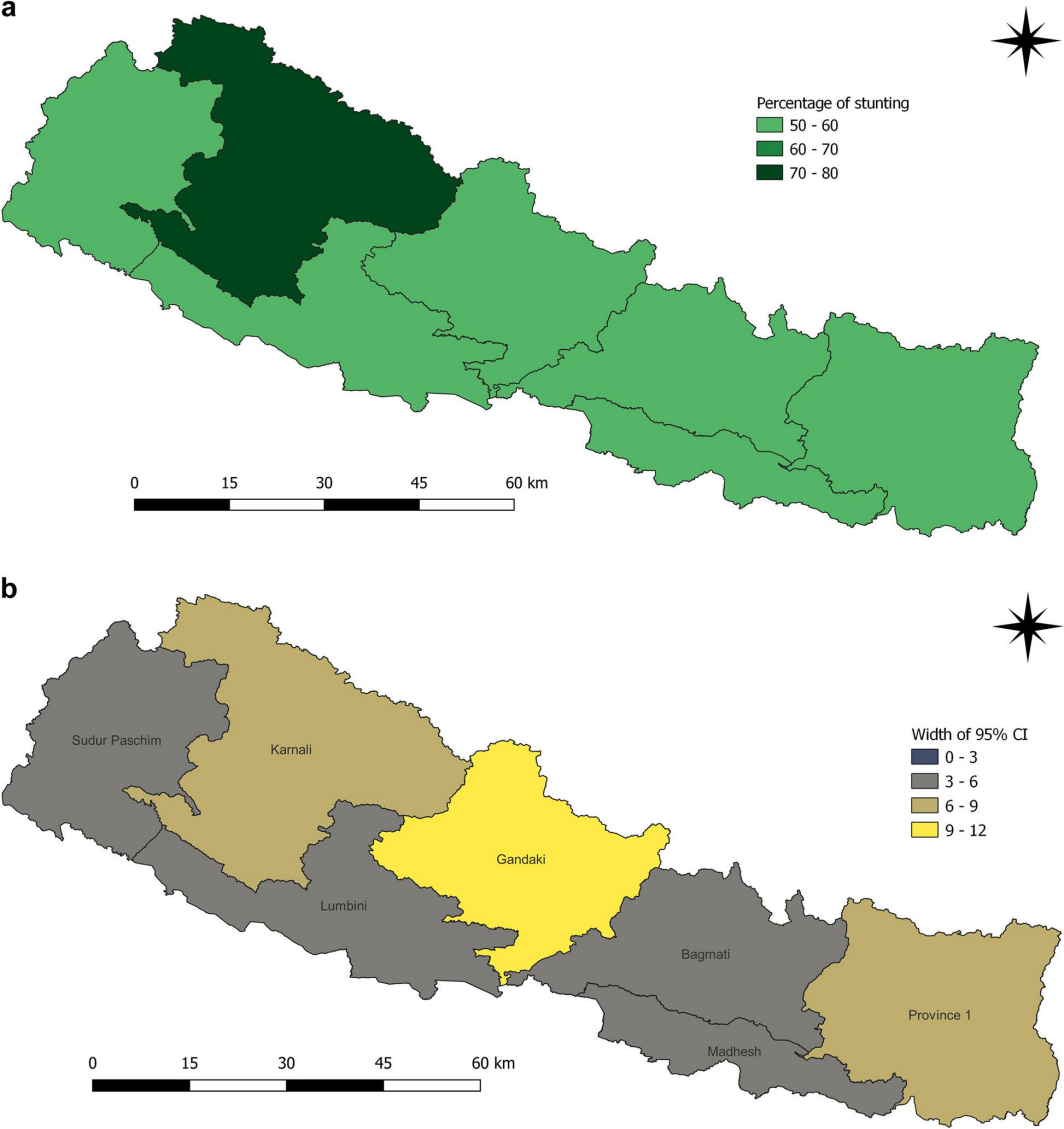
**a** and **b** Prevalence of stunting and width of 95% CI among children under five years as per provinces in 2001

**Fig. 7:**
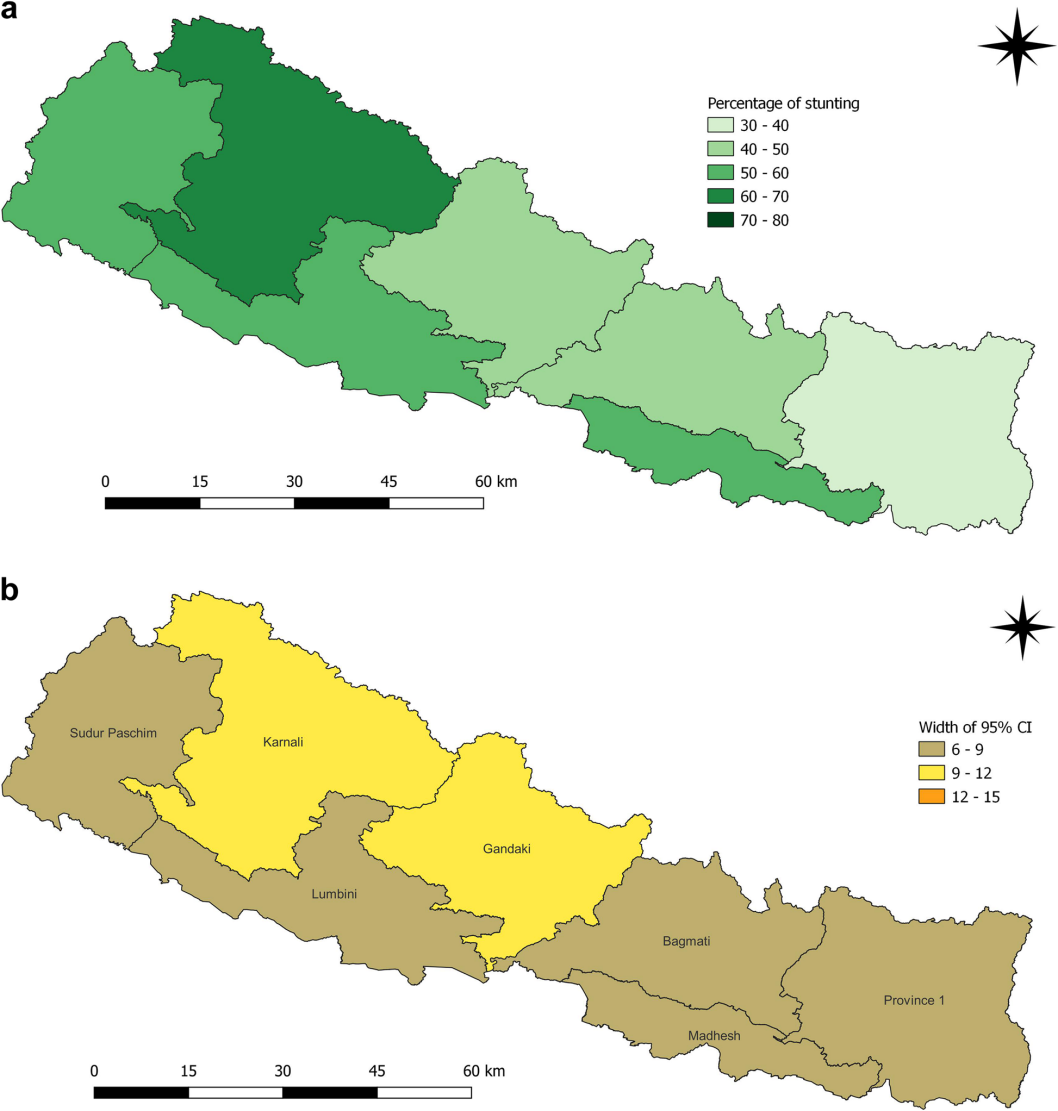
**a** and **b** Prevalence of stunting and width of 95% CI among children under five years as per provinces in 2006

**Fig. 8:**
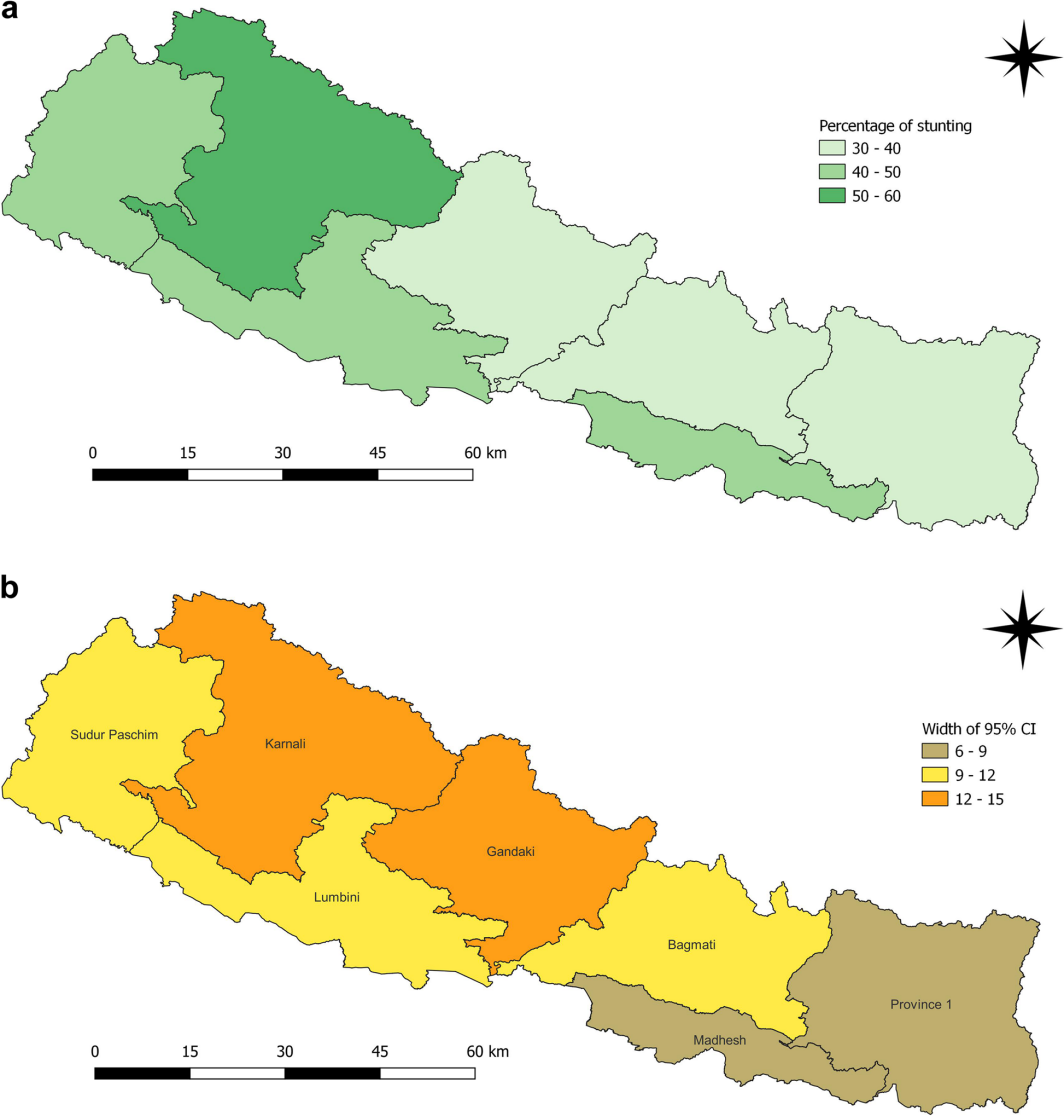
**a** and **b** Prevalence of stunting and width of 95% CI among children under five years as per provinces in 2011

**Fig. 9:**
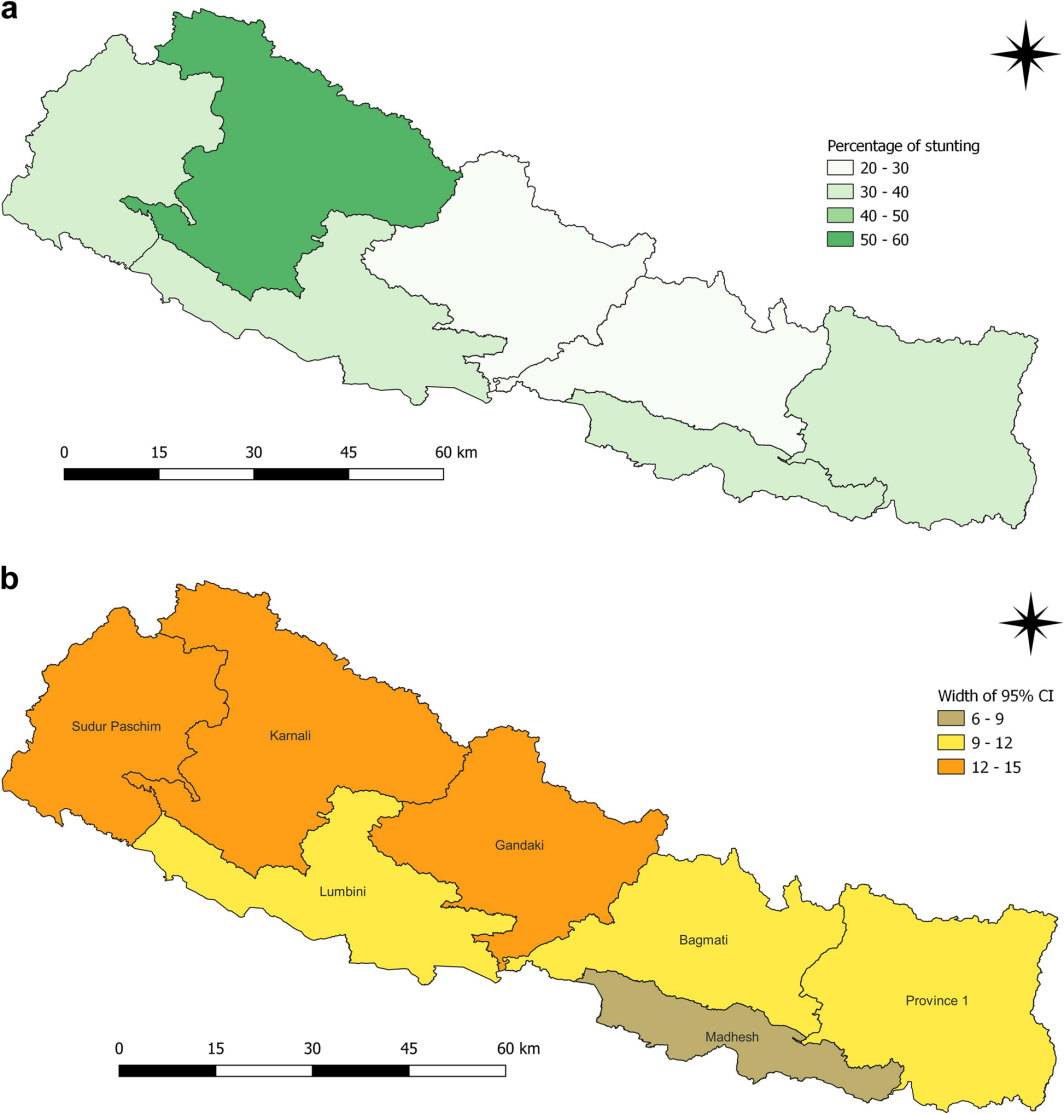
**a** and **b** Prevalence of stunting and width of 95% CI among children under five years as per provinces in 2016

**Fig. 10:**
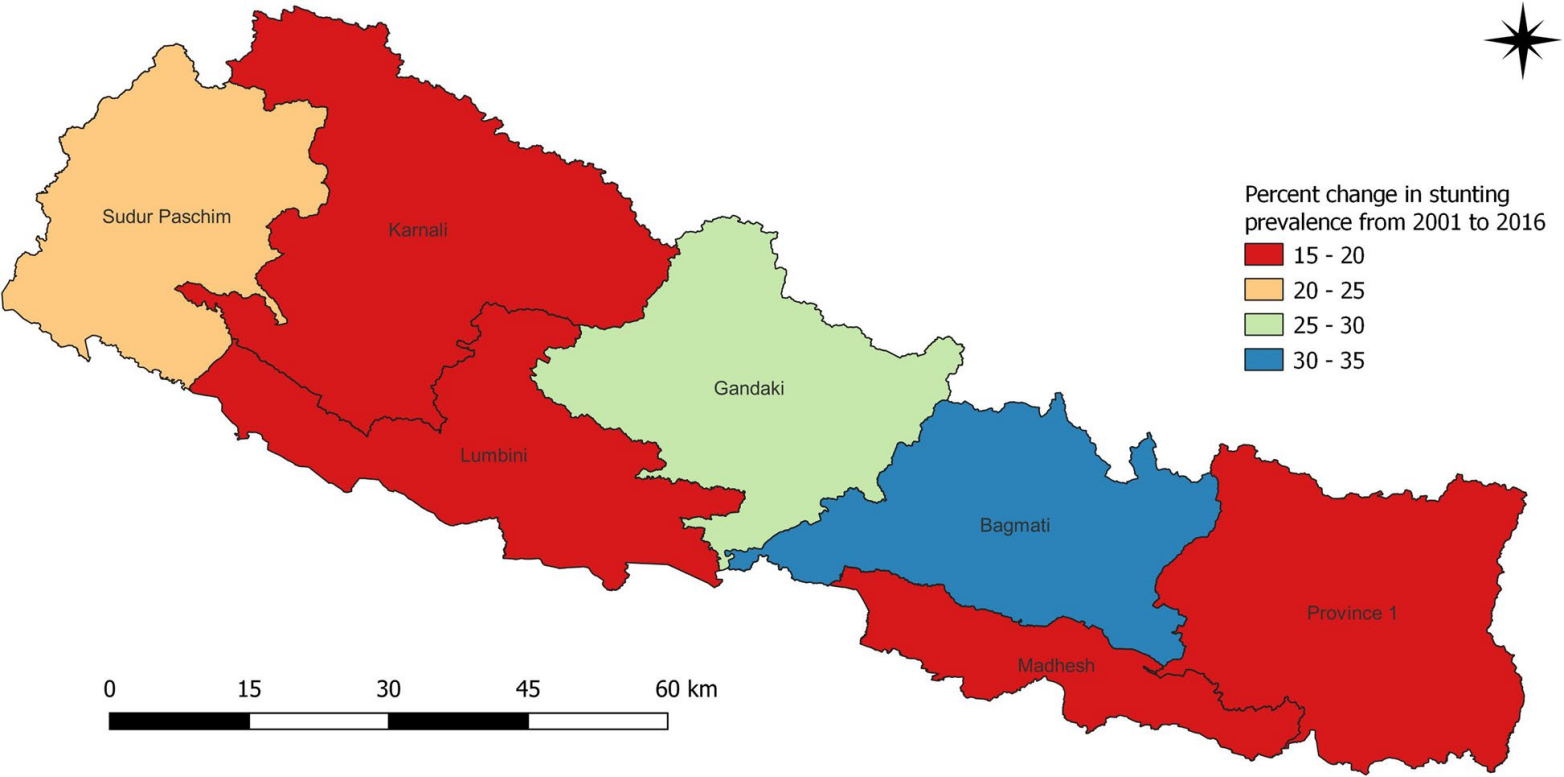
Percent change in prevalence of stunting among children under five years as per districts from 2001 to 2016

The prevalence of stunting is found highest in Karnali Province for all subsequent survey years (Fig. 6a and b). The survey of 2001 reported 2 in 3 or 70.5% children under five years were stunted in Karnali (Table 1). With a reduction by 16% over 15 years, it leveled at 54.4%, but still half of the children under five years were stunted. Besides, Sudur Paschim and Madhesh provinces also had the high proportion of stunted children from 59.9% to 2001 to 36.0% in 2016 and from 56.6% to 2001 to 36.9% in 2016 respectively. The lowest prevalence of stunting among children was noted for Province 1 in 2006 (Fig. 7a and b), Bagmati province in 2011 (Fig. 8a and b) and Gandaki province in 2016 (Fig. 9a and b). The percentage reduced in prevalence of stunting in Province 1, Madhesh, Bagmati, Gandaki, Lumbini and Sudur Paschim provinces were 17.5%, 19.7%, 30.2%, 28.0%, 17.3% and 23.9% respectively (Fig. 10). Therefore, the highest reduction was noted for Province Bagmati by 30.2% and Gandaki by 28%. Figure 10 provides information about the percent change in prevalence of stunting among children under five years from 2001 to 2016.

**Table 1:** Prevalence of stunting and 95% CI among children under five years by seven provinces of Nepal from 2001 to 2016

**Provinces**	**2001**	**95% CI**		**2006**	**95% CI**		**2011**	**95% CI**		**2016**	**95% CI**	
	**Percentage**	**Lower**	**Upper**	**Percentage**	**Lower**	**Upper**	**Percentage**	**Lower**	**Upper**	**Percentage**	**Lower**	**Upper**
Province 1	50.2	46.2	52.7	38.6	34.8	41.4	37.3	32.2	41.2	32.7	27.5	37.2
Madhesh	56.6	54.6	59.3	52.1	48.7	55.0	40.2	36.1	44.0	36.9	33.1	40.8
Bagmati	59.6	57.3	63.2	46.5	41.2	48	34.2	27.7	38.1	29.4	24.5	34.2
Gandaki	54.9	48.8	59.5	47.3	41.5	51.3	36.2	30.9	44.5	26.9	21.9	35.5
Lumbini	55.8	52.3	58	53.2	50.6	57.1	41.8	38.0	47.8	38.5	32.9	42.3
Karnali	70.5	67.3	74.1	62.5	59.5	69.7	55.6	50.5	62.9	54.5	52.0	66.1
Sudur Paschim	59.9	56.9	62.9	52.6	47.5	54.9	46.4	46.6	57.3	36.0	32.3	44.7

Children who slept in the household the night before the survey and who have complete information on date of birth were selected for analysis for the years 2001, 2006 and 2011. For 2016, children who slept in the household the night before the survey only were selected. Data weighted according to DHS recommendations [5]

**Fig. 11:**
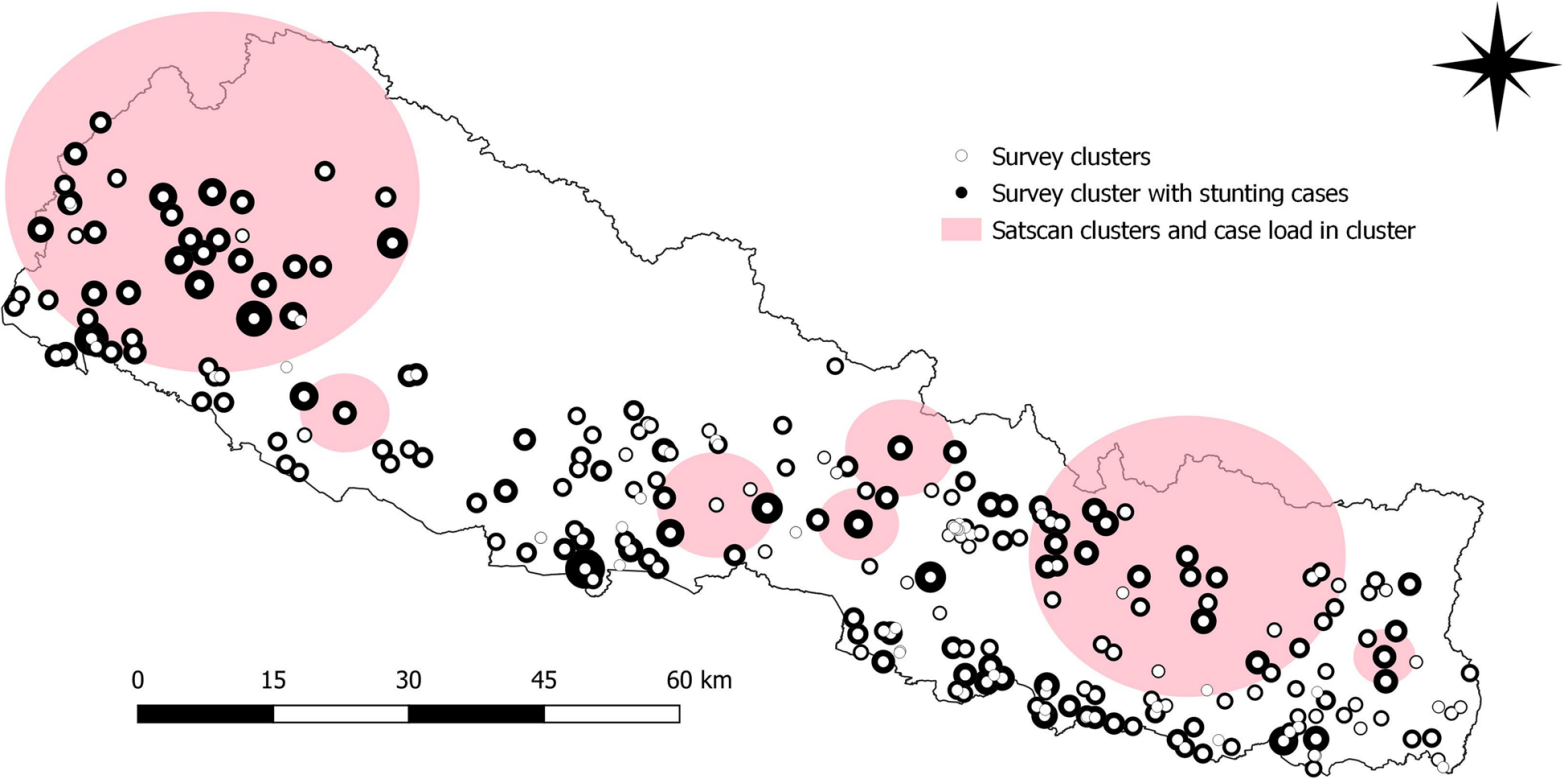
Satscan clusters and stunting caseload in clusters in 2001

**Fig. 12:**
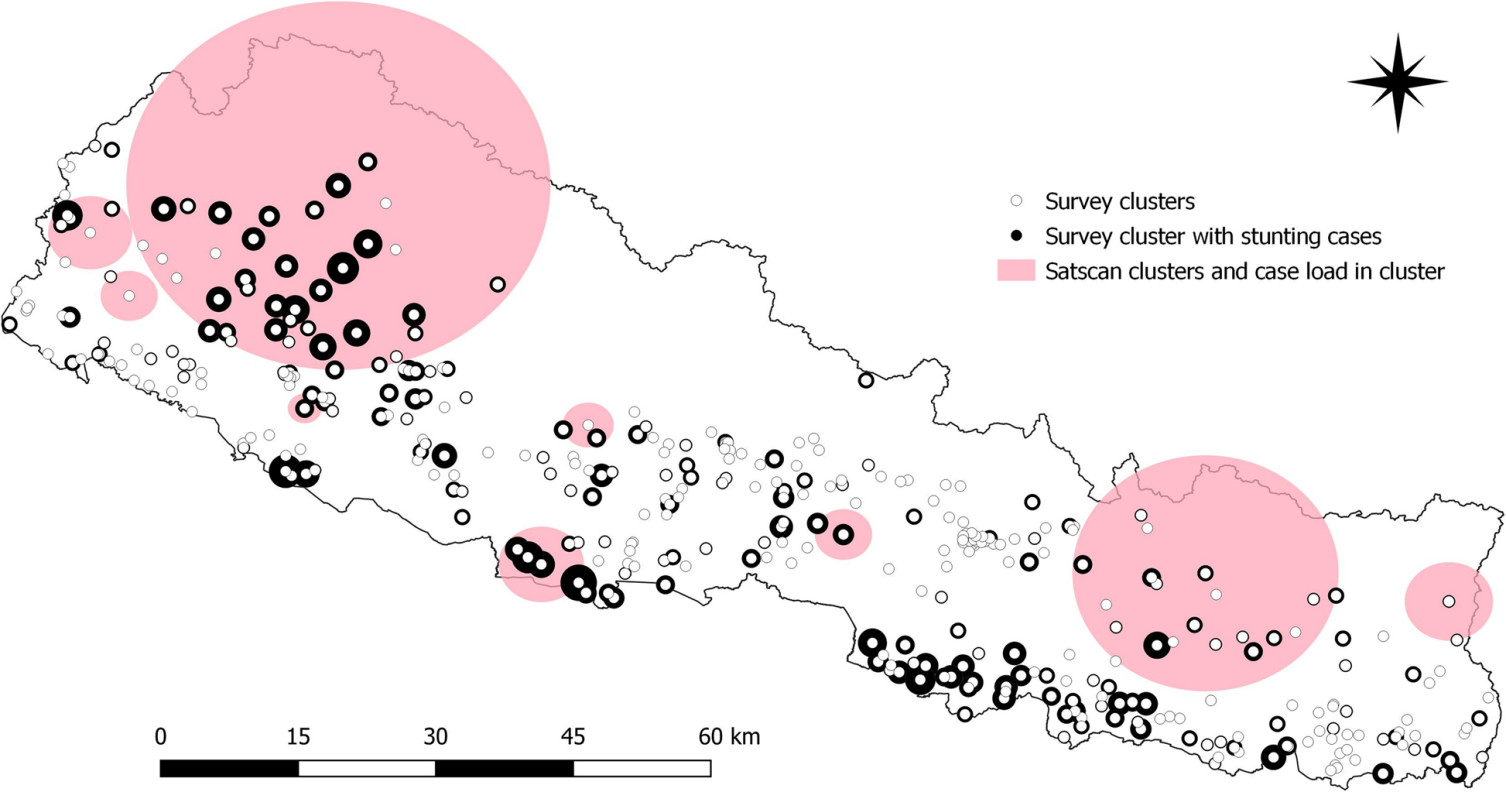
Satscan clusters and stunting caseload in clusters in 2016

Figures 11 and 12 provides information about the clusters identified by satscan and stunting case load in clusters for the year 2001 and 2016 respectively. The radius of the survey cluster with stunting cases is proportional to the number of stunting cases in the cluster. There is no overlap in the satscan clusters for both survey years (2001 and 2016). There were 251 and 383 satscan survey clusters in 2001 and 2016 respectively. In 2001, the average population over time was 24,407 and the total number of cases were 3,698. The annual cases are 15,161.3 per 100,000. The average population over time is 46,383 and the total number of stunting cases is 906 in 2016. The annual cases are 1,949.3 per 100,000.

## Discussion

The reduction in the prevalence of stunting among children under five years is evidence towards Nepal’s effort to improve child health and build a healthier nation. The prevalence of stunting among children in Nepal is comparable to India and Pakistan, countries with similar context. The prevalence of stunting among children in India and Pakistan were 38% [2] and 37.6% [3] as per respective DHS reports of 2015/16 and 2017/18. However, the prevalence of stunting among children is lower in Bangladesh (28%) than Nepal [4]. Many interventions related to improving the nutritional status of children were implemented in Nepal to overcome childhood stunting, wasting and underweight such as Multi-Sectoral Nutrition Plan (MSNP) [15]. In addition to this, other health interventions have been implemented in Nepal to significantly improve maternal and child health by increasing community people’s, especially mother’s accessibility and availability to health services (such as immunizations, vitamin A supplementation, deworming, prenatal, institutional delivery, neonatal and postnatal care) [1, 16]. More specifically, nutrition-specific interventions, which work closely with children and mothers [17] and nutrition-sensitive interventions, which promote agriculture, health, and alternative livelihoods [18] were largely laid out throughout the country. The Government of Nepal has been implementing MSNP incorporating various aspects of nutrition such as water, sanitation and hygiene, education, agriculture, which is now towards the end of the second phase and planning for the third phase has started. The Ministry of Agriculture and Livestock Development (MOALD) sets targets to reduce undernutrition by improving agricultural systems [19]. MOALD has formulated Nepal’s Agricultural Development Strategy for 2015–2035 and Food and Nutrition Security Plan of Action, both prioritizing efforts to increase access to and availability of nutrient-rich foods for improving child and maternal nutritional status [19]. Basically, the interventions of nutrition-sensitive and nutrition-specific sectors should complement each other. A smooth intersectoral coordination within seven sectors of MSNP, integration of thematic areas and adoption of a bottom-up approach will result in better execution of the MSNP. The realization of ownership of improving nutrition by nutrition-sensitive sectors such as agriculture, livestock, education, environment is important. Apart from this, the reduction in the prevalence of stunting among children under five years in Nepal is also determined by improvement in maternal education, economic status, sanitation and hygiene, school meal program, and access to various health services, which have been described in the previous study done by Nepali et al. [1].

The spatial analysis done through QGIS provided an overview of districts and provincial-wise trends of stunting among children under five years for the subsequent survey years. When the average prevalence was disaggregated, they were high for some provinces such as Karnali and Sudur Paschim and low for Province 1 and Gandaki. Similar to this, Jumla, Humla, Dolpa, and Kalikot districts from Karnali province had the highest prevalence of stunting for all survey years. As mentioned above, a continuous effort from the government and contribution from the non-government sector to improve child health has been ongoing, however, the prevalence of stunting has increased over the time of fifteen years for Jumla, Kalikot, Dolpa districts from Karnali province and Gorkha district from Gandaki province. The reduction in the prevalence of stunting has not been remarkable for Madhesh province as well. This is clearly evident from the spatial trend analysis shown by high-resolution provincial and district maps, which is one of the strengths of this study. The provinces and districts that have decreased or increased prevalence or those unchanged from 2001 to 2006 to 2011 and to 2016 are visibly indicated by color gradient followed by its legend. This calls for the allocation of resources and investment proportional to districts and provinces with high prevalence. Such would be a wise use of resources. A targeted and multisectoral intervention in districts and provinces with high prevalence is a requirement.

Alongside, an understanding of factors contributing to stunting at both district and province levels is important while designing interventions. For instance, agriculture and food production is highly affected by geographical features of the area such as availability of the water source for irrigation, dryness of the land, soil fertility, climate, storage facility etc. [20]. The geographical location directly or indirectly controls food security, particularly due to food production through the agriculture system [21]. At the community and household level, people should be encouraged and provided with good skills for practicing agriculture. The local crops of certain areas or districts should be promoted for production, consumption at the household level, and school meal program within the locality and acknowledged as a national asset. The local people should be made aware of its different food recipes and nutritional value. In acknowledgment to this, the intervention to reduce undernutrition should progress from supplement-based and ready-to-use therapeutic foods to food-based interventions, which will bring a long-term solution towards ending hunger and undernutrition among children under five years. Government should also adopt different food fortification measures. In Nepal, terai or plain land is the food house of the whole nation due to abundant food production [22] while the hygiene and health indicators are relatively low for Terai such as high prevalence of diarrhea among children [4]. Food insecurity is higher in mountainous (61.6%) and hilly (53.2%) districts due to low food production contributing to higher undernutrition among children [4]. To bring about positive changes in child nutritional status, the planning for investment of resources should take into account the present status of health, hygiene, education, agriculture. The investment should be made in relation to the status of the multiple sectors such as in terai, more investment is essential towards sanitation and hygiene and less in agriculture and vice versa for hilly regions. Consequently, the provincial government as well as local government should not replicate the intervention packages proposed by the federal and international levels. The intervention should be critically designed based on evidence, past learning and, as per the requirement of the community or district, or province for the betterment of the community people.

One of the major strengths of this study is presenting the trend of stunting as per seven provinces from 2001 to 2016. Since the provincial government has the role and responsibility towards formulating their provincial plans and policies and are accountable for improving child health, this information is highly useful for them because no such information has been published after 7 federal provinces were formed in 2015 in Nepal. The other strength of this study would be that this study is based on the 4 large nationally representative population and large sample sizes warrant a high precision of the findings. The result of this study is based on a cross-sectional study is the limitation. Apart from this, when the national sample size for children under five years was separated into districts, few districts had small sample sizes.

## Conclusion

The inequalities in childhood stunting persisted at district and provincial level although a good decline was noted at the national level. This calls for rigorous attention to be provided to districts (Jumla, Kalikot, Dolpa, Gorkha) and provinces (Karnali, Sudur Paschim, Madhesh) with a high prevalence of stunting. Therefore, the targeted intervention is an appropriate approach rather than blanket coverage. The intervention to reduce malnutrition should progress from nutrition-specific such as supplement based to nutrition-sensitive such as food-based interventions, which will only bring a long-term solution towards ending hunger and undernutrition among children under five years. Also, the provincial government as well as local government should not replicate the intervention packages proposed by the federal and international levels. The intervention should be critically designed based on evidence, past learning and, as per the requirement of the community or district, or province for the betterment of the community people.

## Data Availability

The data that support the findings of this study are available from the DHS website freely (https://dhsprogram.com/data/) but restrictions apply to the availability of these data. Data is however available upon reasonable request noting the concept and objective of the study. Anyone can register and request for the data. The DHS program holds the rights to data.

## Abbreviations

CI: Confidence Interval
DHS: Demographic Health Surveys
GPS: Global Positioning System
MOALD: Ministry of Agriculture and Livestock Development
MSNP: Multi Sectoral Nutrition Plan
NDHS: Nepal Demographic Health Survey
QGIS: Quantum Geographical Information System
SD: Standard Deviation
WHO: World Health Organization

## Declarations

Similarly, the Independent Review Boards of New Era and ICF Macro International reviewed and approved all the data collection tools and procedures for NDHSs. The survey participants were informed about the voluntary nature of participation and confidentiality was assured. Moreover, informed consent was obtained prior to the interview as well as details of the survey were explained to each participant. In the case of children, mothers or caretakers provided consent. All the data enumerators signed the consent form and recorded the consent. Independent Review Boards of both ICF Macro International and New Era approved this consent procedure. The details are mentioned in the NDHS reports.
